# Annotations

**Published:** 1892-02-27

**Authors:** 


### Annotations.

**The National Drink Bill.**

Dr. Dawson Burn has done well to present the British Nation once more with its annual Drink bill. For though the bill is paid and duly receipted, it is a kind of document which should be framed and hung up in some convenient place for frequent reference. The sum spent in strong drink by the people of these islands during the twelve months of 1891 was very considerable, amounting as it did to one hundred and forty-one millions and a quarter sterling. The statement is almost as difficult to believe as an ecclesiastical miracle. It is, however, true, and we may therefore consider it in detail with some advantage. This grand total, divided by the number of the population, gives £3 15s. worth of beer, wine, and spirits, for every man, woman, and child, in the United Kingdom, or £18 15s. spent by every head of a family of five persons. Let us admit that beer, wines, and spirits, in proper quantities, are useful things. But when that is admitted, we must also grant that in excess they are dangerous poisons. If we exclude children, we have left an average expenditure of about four shillings a-week for every man and woman of adult age. Now a pint of beer a-day, or its equivalent in wine and spirits, is as much as the ordinary man or woman can use with safety to health, and the cost of a pint of beer is threepence, and the cost of seven days' pints of beer is one-and-ninepence. Even if there were no total abstainers to be deducted, the whole population is thus shown to be drinking at least twice as much as it should. But it is possible that a third of our adults are abstainers; and a third of the consumed alcohol is therefore to be added to the quantity drunk by only two-thirds of the population. It may be taken for granted that one of these two-thirds drinks no more than is compatible with health; and therefore the third that is left must take at least three or four times more than its proper quantity. In other words, a full third of our people are destroying their livers, kidneys and brains with alcohol, and laying up a fine reserve of dropsies, heart diseases, dyspnœas, mental incapacities, and general miseries for the last half-dozen years of their lives. The doctor in a man is alarmed by this state of things, and the philanthropist in him is profoundly grieved. Medical men have done much to enlighten the public upon the scientific aspect of the question; and total abstainers, if somewhat fanatical in their methods, have urged home the moral factors. One cannot but ardently desire that thinking people will lay these facts and scientific deductions to heart, and will, each in his own place, act upon them. The late Dr. Magee said he would rather see England free than sober. But, as a matter of fact, neither England nor any other country can be free unless it is sober.

**Professional Witnesses.**

Professional witnesses, more especially if they belong to any branch of the healing profession, often bring the blush of shame to the faces of their fellow professionals. Veterinary surgeons have no reason to be proud of the performance of one of their fellows who appeared as a witness before Mr. Alderman Renals at the Guildhall a few days ago. A carman of the name of Amonda was charged with cruelly working a horse whilst lame. Luigi Fraulo, provision merchant, was brought up for causing the horse to be so worked. Mr. Savournin, veterinary surgeon, stated that the sores on the animal were very bad, and had not been attended to. Mr. J. Baxter, M.R.C.V.S., was called for the defence, and stated that the horse was fit to work in its present condition. Alderman Renals thereupon delivered himself in the following terms: "I know something about horses, but I never heard such a statement from a veterinary surgeon. I shall fine you, Amonda, 40s. for taking the horse out, and the owner £5, and costs, or a month, and if he comes here again he will assuredly go to prison. I wish to say that as long as I sit on this bench I hope I shall not hear such evidence as one veterinary surgeon has given. It is a disgrace to the profession." There are no doubt always two sides to every case, and it is quite fair that both should be stated. It seems, however, to be considered by professional witnesses that if one side be black, the other must necessarily be white, or at least that it can be made to appear white by sufficiently hard swearing. It further appears to be considered professionally proper to swear as hard as the necessities of the case demand, provided a witness is duly employed and his fees paid. This is of a piece with the behaviour of lawyers in court and elsewhere. Their business is, like that of other professional persons, to make the side which employs them triumphant at all costs. Well, if we must hear these things we must. Rome was not built in a day, and it is plain that simple truth and honest facts are not to prevail in a day. But professional people, being generally better educated than other persons, ought to have a little more regard for things as they really are, and ought not to expose themselves to such strictures as Alderman Renals thought proper to inflict upon Mr. Baxter. It is evident that Mr. Baxter's testimony did the defendant more harm than good, and that the result to Mr. Baxter himself was anything but creditable. Some day, no doubt, public opinion will be strong enough to put an end to the extraordinary doings of expert witnesses, and thus to give the simple facts of the case a fair field and no favour.

**Physiologists and Small Holdings.**

Every English physiologist who has more than a histological acquaintance with his fellow creatures will be glad that there is at length some prospect of a Small Holdings Act being passed, and of the re-marriage of the sons of the soil to their early love. For though science does not know much of country, or narrow patriotism, yet we do not think the more of an English scientist if he regards England and his fellow Englishmen with supreme indifference. If we are to continue to produce capable men for our markets, our law courts, our pulpits, and our consulting-rooms, as well as for our armies and navies, we must produce individuals of masculine physical build and sound constitution. We might as well attempt to improve the breed of racehorses by breeding in London mews and training in hansoms and four-wheelers, as hope to improve the race of human beings by breeding and bringing them up in the streets and cabined dwelling-houses of large towns. The countryman has the advantage of the town-bred man both in physique and in mental capacity. In spite of sundry drawbacks in his early education, he beats the town man when he comes up to town on the latter's own ground; and that not only in muscle, and in physical staying power, but in intellectual breadth and mental tenacity. The true Englishman, who believes in the capacity and the virtue of his race, desires, as one of his chief wishes in life, to see the number of stalwart, enduring, just, brave, and loyal Englishmen greatly increased. The country is the place for such increase, not the streets of towns. No town-bred man ever loved his street or his numbered house as the countryman loves his home, his orchard, his meadows and cornfields, and the woods and hills that shut him in from the great world outside. Men and women bred in such circumstances as these know what home is: they love the roof that protected them in childhood, and they would fight for it to the death if need were. What we ought to do in this country is to multiply homes of this kind by tens of thousands: to multiply them, and to give each steadfast and resolute Englishman an opportunity of making such a home his own. People tell us that the labourer has no capacity for self-denial, and that he will never become even the smallest of proprietors except in the rarest of instances. This may be true to some extent of the Southern labourer. But the case is very different with the men of the Midlands and of the North of England and Scotland. Give but these a chance, and they will become proprietors in their tens and hundreds of thousands. They will form for the country a backbone of sober and reasonable liberal-conservatism such as it never had before. Besides, there are thousands of farmers' sons who are not able in early life to enter upon considerable farms because their capital is not sufficient. These drift to large towns, and lose caste and self-respect by becoming cabdrivers, tramway conductors, and the like. They would much rather be masters of their own small freeholds than servants of other men, even at high wages. This spirit of freedom and manhood our statesmen should religiously conserve. A country can only be strong when its people are strong, and great when its freemen are great. We have now an opportunity of entering upon a new and nobler phase of national life. Let us hope that differences will be sunk and that patriotism will prevail to give us a united and mighty impulse in the new direction.

